# Current Use and Barriers to POCUS in Women's Health: A National Survey of Veterans Affairs Medical Centers

**DOI:** 10.24908/pocusj.v11i01.19484

**Published:** 2026-04-22

**Authors:** Megha Gupta, Sarah Hanson, Stephen Wagner, Amir A. Shamshirsaz, Angela Ranzini, Michael J. Mader, Nilam J. Soni

**Affiliations:** 1 Department of Obstetrics and Gynecology, Beth Israel Deaconess Medical Center, Department of Obstetrics, Gynecology and Reproductive Biology, Harvard Medical School, Boston, MA, USA; 2 Division of Maternal-Fetal Medicine, Department of Obstetrics and Gynecology, Texas Children's Hospital, Baylor College of Medicine, Houston, TX, USA; 3 Division of Maternal Fetal Medicine, Department of Obstetrics and Gynecology, MetroHealth System, Case Western Reserve University, Cleveland, OH, USA; 4 Research Service, South Texas Veterans Health Care System, San Antonio, TX, USA; 5 Medicine Service, South Texas Veterans Health Care System, San Antonio, TX, USA; 6 Division of Hospital Medicine, University of Texas Health San Antonio, San Antonio, TX, USA; 7 Division of Pulmonary Diseases & Critical Care Medicine, University of Texas Health San Antonio, San Antonio, TX, USA

**Keywords:** Maternal point-of-care ultasound, POCUS, Obstetrics, Barriers, Training

## Abstract

**Background:**

Point of care ultrasound (POCUS) has become an invaluable tool in healthcare across multiple disciplines. For the past 30 years, obstetricians and gynecologists in many hospitals have had access to ultrasound equipment and commonly utilize it in labor and delivery suites and emergency rooms for a broad range of conditions. POCUS can be divided into gynecologic and obstetric applications—the latter can be further categorized into fetal POCUS and maternal POCUS. While fetal POCUS primarily assesses the fetus, maternal POCUS is crucial for evaluating conditions that impact a mother's health during pregnancy, intrapartum, and postpartum, such as cardiopulmonary status. The COVID-19 pandemic highlighted the importance of maternal POCUS for timely diagnosis and management, which can ultimately reduce maternal morbidity and mortality. This study aimed to characterize the current use of maternal POCUS, identify barriers to its adoption, and explore opportunities for greater integration into clinical practice nationally among Women's Health (WH) departments within the Veterans Affairs (VA) healthcare system.

**Methods:**

A prospective observational study was conducted from June 2019 to March 2020 through a web-based survey distributed to all VA medical centers. The survey was first distributed to all chiefs of staff about facility-level POCUS use, training, competency, and policies. A follow-up survey was sent to all WH chiefs to obtain service-level data on diagnostic and procedural POCUS use, training needs, workflows, and equipment availability. Statistical analysis utilized the Chi-squared test to uncover associations between POCUS use and various group characteristics, with a significance threshold of p<0.05.

**Results:**

Response rates were 100% among chiefs of staff (n = 130) for the facility-level survey and 77% among WH chiefs (n = 61) for the service-level survey. Diagnostic or procedural POCUS was used by only 30% of all WH groups. The most frequently reported diagnostic applications included assessment of the uterus (23%), ovaries (23%), and intrauterine pregnancy (16%). The most frequently identified procedural application identified was intrauterine device insertion, reported by 23% of the groups. Key barriers to POCUS use included a lack of equipment (56%), lack of trained clinicians (49%), insufficient funding for training (28%), and lack of support staff (26%). While 69% of WH chiefs expressed support for POCUS training, only 23% of chiefs reported having a structured process for obtaining POCUS training for their clinicians. This discrepancy underscores the need for enhanced education and awareness initiatives to align clinician perspectives with the growing benefits of POCUS in clinical practice.

**Conclusion:**

This national survey highlighted the low utilization of maternal POCUS among WH clinicians in the VA healthcare system and pointed to critical barriers, including equipment shortages and training gaps. Addressing these barriers through enhanced training, resource allocation, and leadership support is essential to fully leverage the potential benefits of POCUS use in maternal care. Future efforts should focus on evaluating the impact of improved POCUS training and investment in POCUS infrastructure on maternal health outcomes.

## Introduction

Since ultrasound was first described in obstetrics and gynecology (OBGYN) in 1958, its primary application has been focused on fetal evaluation with minimal emphasis on peripartum maternal care [1]. By the 1990s, ultrasound equipment was widely available in labor and delivery suites and was utilized to assess a variety of obstetric conditions. In recent years, particularly during the COVID-19 pandemic, the untapped utility of point of care ultrasound (POCUS) in maternal care (maternal POCUS) has been increasingly recognized [2,3]. Maternal POCUS is a focused bedside ultrasound exam performed by a clinician caring for a mother. It can rapidly evaluate maternal conditions related to pregnancy that are not included in traditional obstetric ultrasound exams, such as cardiopulmonary status [4]. With the rising incidence of cardiopulmonary disorders that lead to maternal morbidity and mortality, there is a compelling need to expand maternal POCUS use [1,5].

Obstetricians and gynecologists (OBGYNs) are uniquely poised to incorporate maternal POCUS into their practices [6]. They receive extensive ultrasound training during residency, have access to ultrasound equipment, and have established standard workflows for documentation and image archiving [7,8]. Maternal POCUS can aid in the prompt diagnosis and management of critical obstetric conditions. If untreated, these conditions can lead to severe complications that pose risks to maternal health, such as pulmonary edema in preeclampsia, reduced cardiac function in peripartum cardiomyopathy, and right ventricular failure in amniotic fluid embolism [9].

Despite the potential benefits of maternal POCUS, few obstetricians currently utilize POCUS beyond traditional obstetrical ultrasound [10]. The current use, training needs, and barriers to maternal POCUS adoption among OBGYNs remain largely unknown. To address this knowledge gap, we conducted a national survey of all Women's Health (WH) departments within the Veterans Affairs (VA) healthcare system to characterize the current landscape of maternal POCUS use and identify opportunities for increased integration into practice. Our findings can guide the implementation of maternal POCUS use in OBGYN, including the development of training and targeted investment in POCUS infrastructure.

## Methods

A prospective observational study of all VA medical centers was conducted between June 2019 and March 2020. A multi-disciplinary POCUS Technical Advisory Group with physicians from emergency medicine, internal medicine, hospital medicine, pulmonary medicine, and critical care medicine collaborated with the VA's Healthcare Analysis and Information Group to develop and disseminate a web-based survey systemwide (Verint Systems, Inc.®, 2019). This study was reviewed by the Institutional Review Board of the University of Texas Health Science Center San Antonio and deemed to be non-research (Protocol Number: HSC20210630NRR).

The survey included questions on current use, barriers, institutional support, equipment, and POCUS training needs—encompassing a wide range of diagnostic and procedural applications categorized by body system (see Supplementary Material S1). Question types were multiple-choice, forced-choice (yes/no), open-ended with numerical or free-text entry, and free-text boxes when “other” was selected. For questions of prevalence, respondents were provided the option to answer as few (1–25%), some (26–50%), many (51–75%), most (76–99%), or all (100%).

The survey was first distributed to all chiefs of staff (n = 130) of VA medical centers nationwide between August and October 2019. The chief of staff survey included ten questions about facility-level POCUS use, training, competency, and policies, and required contact information for all WH chiefs. Next, a follow-up survey with 18 questions was sent to all WH chiefs (n = 79) to obtain service-level data on diagnostic and procedural POCUS use, training needs, workflows, and equipment availability (Supplementary Material S2). The survey period for WH chiefs started in December 2019 but ended early in March 2020 due to the COVID-19 pandemic.

Current POCUS use and training desired were averaged across all diagnostic and procedural applications when reported by body system or category. We used the Chi-squared test to determine associations between POCUS use and group characteristics. A p-value <0.05 was considered statistically significant.

## Results

All chiefs of staff (n = 130) of VA medical centers completed the facility-level survey, achieving a 100% response rate. In contrast, 61 WH chiefs completed the service-level survey (77% response rate). Most WH groups were located in large, urban medical centers with high complexity ratings and had a median of 80 hospital beds (IQR 52–110) (Table 1).

**Table 1.** Characteristics of VA Medical Centers per WH Chiefs. VA, Veterans Affairs; WH, women's health; POCUS, point of care ultrasound.

Characteristic	N = 61 (%)
**Active Primary Care Patients**	
<5,000	21 (34%)
5,000–10,000	27 (44%)
>10,000	13 (21%)
**Total Hospital Beds at VA medical centers**	
<50	15 (25%)
50–100	26 (43%)
>100	20 (33%)
**Veterans Health Administration Facility Complexity Level** ^1^	
High	53 (87%)
Low	8 (13%)
**Region**	
Northeast	11 (18%)
Midwest	14 (23%)
South	25 (41%)
West	11 (18%)
**Location**	
Urban	58 (95%)
**≥1 WH clinician uses POCUS**	18 (30%)
**Clinicians have desire for POCUS training**	20 (33%)
**Service Chief knows ≥1 facility-level POCUS policy**	22 (36%)
**Process to obtain POCUS training**	14 (23%)
**Service Chief supports POCUS training**	42 (69%)

1 High complexity facilities have high levels of patient volume, patient risk, specialists, teaching, and research. Low complexity facilities have medium to low levels of patient volume and patient risk, and some to little teaching or research.

### Current use

**Figure 1.**
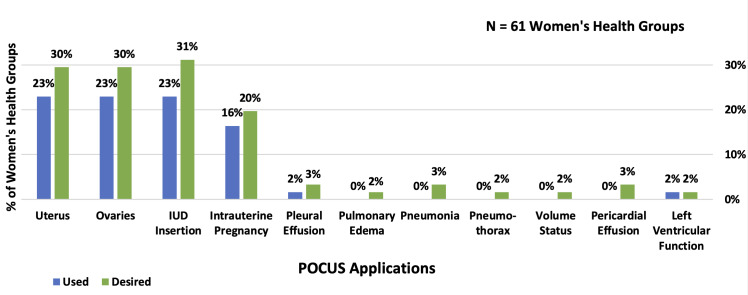
Common Point of Care Ultrasound (POCUS) Applications Used and Training Desired in Women's Health (WH) Groups.

Diagnostic or procedural POCUS was reported as being used by at least one WH physician in 30% of WH groups. The most common diagnostic applications included assessment of the uterus (23%), ovaries (23%), and intrauterine pregnancy (16%). The most frequently reported procedural application was intrauterine device insertion, cited by 23% of WH groups (Table 2 and Figure 1). There was no reported use of maternal POCUS for critically ill patients.

**Table 2.** Current POCUS Use and Desire for Training per WH Chiefs (N = 61). POCUS, point of care ultrasound; WH, women's health.

POCUS Application	Current Use N = 61 (%)	Desire for Training N = 61 (%)
***Gynecological***		
Uterus	14 (23%)	18 (30%)
Ovaries	14 (23%)	18 (30%)
Intrauterine Device Insertion	14 (23%)	19 (31%)
***Obstetrical-Fetal***		
Intrauterine Pregnancy	10 (16%)	12 (20%)
***Obstetrical-Maternal***		
Pleural Effusion	1 (2%)	2 (3%)
Pulmonary Edema	0	1 (2%)
Pneumonia	0	2 (3%)
Pneumothorax	0	1 (2%)
Volume Status (Inferior Vena Cava/Internal Jugular)	0	1 (2%)
Pericardial Effusion	0	2 (3%)
Left Ventricular Function	1 (2%)	1 (2%)

### Barriers

The most commonly reported barriers to POCUS use among WH groups were lack of equipment (56%), insufficient number of trained clinicians (49%), limited funding for training (28%), and inadequate number of support staff (26%) (Table 3).

**Table 3.** Barriers to POCUS Use in WH Groups per Chiefs.

Barrier	Number of Women's Health Groups N = 61 (%)
**TRAINING**	
Lack of Trained Clinicians	30 (49%)
Lack of Funding for Training	16 (26%)
Lack of Training Opportunities	13 (21%)
Lack of Funding for Travel	8 (13%)
**At least one of the TRAINING Barriers listed above**	**35 (57%)**
**EQUIPMENT**	
Lack of Ultrasound Equipment	34 (56%)
Lack of Funding for US equipment	15 (25%)
**At least one of the EQUIPMENT Barriers listed above**	**34 (56%)**
**INFRASTRUCTURE**	
No Clinician Champion	17 (28%)
Lack of Funding for Support Staff	17 (28%)
Lack of Funding for Simulation Space	14 (23%)
Lack of Privileging Criteria	10 (16%)
Lack of Standard Reporting Form	11 (18%)
Lack of Facility Leadership Support	4 (7%)
Lack of Image Archiving	11 (18%)
**At least one of the INFRASTRUCTURE Barriers listed above**	**30 (49%)**
**OTHER**	
No Perceived Benefit	11 (18%)
No Barriers Identified	11 (18%)

### Training

Training remains a critical barrier for POCUS use in dedicated WH groups at VA medical centers. Among the WH chiefs surveyed, 49% described encountering at least one training-related barrier. The most frequently cited barrier was the lack of trained clinicians, as reported by 49% of chiefs. Although only 33% of WH chiefs felt their clinicians desired POCUS training, 69% of chiefs indicated they would support sending their clinicians to a VA POCUS training course. This reflected a strong commitment to enhancing clinician training in POCUS. Conversely, only 23% of chiefs reported having a structured process for obtaining POCUS training for their clinicians. Additional training-related barriers included insufficient funding for training (26%), lack of training opportunities (21%), and inadequate travel funding (13%). These further underscored the need for institutional investment in training to standardize POCUS implementation.

### Infrastructure

Infrastructure-related barriers were also reported as significant challenges to POCUS adoption in WH groups. Forty-nine percent of chiefs reported at least one infrastructure barrier, with lack of clinician champions and funding for support staff cited by 28% of chiefs. Additional barriers included insufficient funding for simulation space (23%), unclear privileging criteria (16%), and the need for standardized reporting forms and image archiving (18%).

## Discussion

Our national survey highlighted both the current landscape of POCUS utilization and significant barriers to its implementation in WH groups within the VA healthcare system. Despite the recognized potential for POCUS to enhance maternal care, our survey revealed that only 30% of WH groups were currently utilizing POCUS. This low adoption rate underscores a critical gap between the capabilities of POCUS and its integration into routine clinical practice by OBGYNs.

To address this gap, it is particularly important to distinguish between gynecological POCUS, which focuses on evaluating the uterus and ovaries, and obstetric POCUS, which is further divided into maternal and fetal applications. Fetal POCUS specifically pertains to the assessment of the fetus and includes standard evaluations, such as confirming intrauterine pregnancy, fetal presentation, fetal number, fetal heart rate, biometry, amniotic fluid volume, biophysical profile, and placental location. In contrast, maternal POCUS involves assessing conditions related to the health and well-being of the mother during pregnancy, encompassing cardiac, pulmonary, abdominal, and lower extremity venous ultrasound. This structured understanding underscores the importance of clarity when discussing POCUS in OBGYN as a specialty, as ambiguity can hinder effective communication and application in clinical settings.

Recognizing the distinctions among POCUS applications highlights a concerning underutilization of POCUS in OBGYN settings. Our survey revealed significant gaps in maternal POCUS use among WH patients. No chiefs reported using POCUS for common clinical scenarios, such as evaluating pulmonary edema in preeclampsia patients on magnesium sulfate, identifying intra-abdominal bleeding or uterine rupture, determining volume status, or ruling out lower extremity deep venous thrombosis. Moreover, 18% of chiefs perceived no benefit from using maternal POCUS, indicating a disconnect between its potential utility and practical application. This may be explained by insufficient awareness or exposure to common maternal POCUS applications that are supported by an ever-increasing body of evidence. Addressing these gaps through case presentations, peer-led workshops, and targeted outreach may enhance awareness, shift perceptions, and increase acceptance of maternal POCUS among OBGYNs [11]. Highlighting the accuracy and manageable learning curve of maternal POCUS in various clinical scenarios can further demonstrate its efficiency and effectiveness, encouraging more OBGYNs to incorporate it into their practices [12,13].

The primary barriers identified—lack of equipment, trained clinicians, and funding for training—reflect health system and institutional challenges that need to be addressed for broad and standardized POCUS adoption across WH settings [14]. Most (56%) WH groups reported significant barriers related to equipment availability. It is unclear whether this refers to availability of machines or specific probe types, or both. Curvilinear probes are generally more accessible, while transvaginal probes require more time for high-level disinfection and patient privacy. This is surprising given that most ultrasound applications are considered standard of care in OBGYN. We suspect these WH groups are obtaining referral ultrasound exams through radiology which cannot be elucidated from our data. Nonetheless, this finding suggests that half of WH groups lack portable ultrasound equipment for various POCUS applications, underscoring the need for health system investment in ultrasound technology to improve women's care overall.

Training has become a major challenge, and nearly half of WH groups reported a lack of trained clinicians. However, considering only 33% of clinicians expressed interest in POCUS training raises concerns about their perception of its relevance and usefulness in their clinical practice. Previous studies have shown high physician interest in learning POCUS, but the low interest in WH POCUS may be due to historical undervaluation of WH. Given this limited interest, integrating POCUS into routine practice may be difficult if it depends only on clinician-initiated training efforts. These findings emphasize the importance of educational initiatives that showcase POCUS benefits and integrating structured training programs within health systems [15,16]. The strong support from 69% of WH chiefs for sending their clinicians to an internal POCUS training course is encouraging and could serve as a foundational step toward enhancing WH clinicians' competency and confidence in using POCUS. In the meantime, utilizing dedicated ultrasound consult services might be an effective alternative to increase access and incorporate POCUS in WH.

Additionally, infrastructure-related barriers, including inadequate clinician champions and insufficient funding for support staff, limit integration of POCUS into WH practices [17]. The presence of clinician champions is important for driving change and fostering a culture that embraces innovation. The reported lack of leadership support in some facilities suggests that engagement from both health system and institutional administration is necessary to allocate appropriate resources to champion POCUS initiatives. Further research is needed to evaluate the effectiveness of specific training programs and resource allocation on the adoption of maternal POCUS. Additional studies should explore the impact of integrating POCUS on maternal health outcomes and identify strategies to increase awareness and acceptance of POCUS among WH clinicians.

Our study had both strengths and limitations. A key strength of our survey was the high response rate (100% for chiefs of staff and 77% for WH chiefs). As well, the data are unique, addressing a gap in published national surveys on POCUS use among OBGYNs. However, an important limitation was our use of “POCUS”—a term that needs clearer definition in the context of WH. Chiefs who completed the survey may have been unclear if the survey referred to obstetric (fetal and maternal) POCUS or gynecological POCUS exams, which may have affected their responses. Additionally, the VA system includes many community-based outpatient clinics, and WH clinics in the outpatient setting were not queried in this survey. Another limitation pertained to the timing of data collection. The survey period for WH chiefs started in December 2019 and ended early in March 2020 due to the COVID-19 pandemic. This early termination may have influenced the responses, and the pandemic may have further impacted POCUS utilization in ways not captured by our survey. These factors may limit the generalizability of our findings.

## Conclusion

Our study revealed relatively low use of obstetrical (fetal and maternal) and gynecological POCUS among WH clinicians at VA medical centers nationally. However, for all POCUS applications, the desire for training exceeded current usage. Addressing critical barriers, such as the lack of equipment and training opportunities, and fostering a supportive infrastructure will be essential steps to realizing the full potential of both OBGYN and non-OBGYN POCUS applications in WH. Future research should focus on evaluating how POCUS training and equipment provision impacts POCUS implementation and maternal health outcomes. This will pave the way for a more proactive approach to POCUS implementation in WH. By leveraging the unique position of OBGYNs who have access to ultrasound equipment, we can advocate for increased maternal POCUS, ultimately aiming to reduce morbidity and mortality associated with cardiopulmonary and other pregnancy disorders.
