# Supplementary material for: Current Use and Barriers to POCUS in Women's Health: A National Survey of Veterans Affairs Medical Centers
Source: POCUS J. 2026 Apr 22;11(1):104–9. doi: 10.24908/pocusj.v11i01.19484 (PMC13161786; doi:10.24908/pocusj.v11i01.19484)
Supplement: Supplementary file 1 [file pocusj-11-01-19484-s001.pdf]

Supplemental File 1: POCUS Survey for Chief of Staff

2019 VHA Point of Care Ultrasound (POCUS) Survey – Chief of Staff

The Office of Patient Care Services periodically surveys the programs it administers to obtain information for stakeholders that is not readily available through other means. The purpose of this survey is to:

1. Assess the current state of POCUS in acute care settings in VHA,
2. Assess POCUS training needs across specialties, and
3. Compare data/explore relationships that may be related to POCUS.

The VHA defines **Point of Care Ultrasound** as:

"Point of Care Ultrasound is defined as a goal-directed, bedside ultrasound examination performed by a healthcare provider to answer a specific diagnostic question or to guide performance of an invasive procedure." – Reference: *Point of Care Ultrasound, 1st edition.*

Respondent: Chief of Staff or designated representative.

Key Instructions:

1. In some cases, one management team is responsible for more than one VA Hospital. The ***Chief of Staff or designee*** should submit one survey response ***<u>for each</u>*** VA Hospital under their area of responsibility.

2. Respondents must identify the Service or Section Chief/Designee who will submit a follow-up survey later in the year for ***<u>each individual</u>*** service or section in existence at your facility, in question nine.

This survey takes approximately 15 minutes to complete but may take additional time to gather the appropriate information.

Provide the name of the individual who should be contacted if clarification of responses is needed.

VISN Number: (Select from list provided: 1 – 23)

Facility Name: (Select from list provided)

Point of Contact information (Person completing the survey)

Name of person completing this survey ________________________Title ____________________

Phone Number (including area code) __________________________Extension _______________

VA Email (user@va.gov) ___________________________________

Point of Care Ultrasound (POCUS) definition: "Point of Care Ultrasound is defined as a goal-directed, bedside ultrasound examination performed by a healthcare provider to answer a specific diagnostic question or to guide performance of an invasive procedure." – Reference: *Point of Care Ultrasound, 1st edition.*

1. Is Point of Care Ultrasound (POCUS) training desired at your facility?
   ( ) Yes
   ( ) No
   If yes,
   a. *For each body system below*, select the **diagnostic and procedural** POCUS **training** modules your staff could benefit from.

Body System	(For each specialty below, you must choose at least one response)	
**a. Cardiac**	[ ] Pericardial Effusion<br>[ ] Left Ventricular Function<br>[ ] Venous Mapping<br>[ ] Volume Status (Inferior Vena Cava (IVC)/Internal Jugular (IJ))	[ ] Advanced Hemodynamic Measurements (e.g., cardiac output, stroke volume)<br>[ ] Pericardiocentesis<br>[ ] None of the above
**b. Pulmonary**	[ ] Pleural Effusion<br>[ ] Pneumothorax<br>[ ] Pulmonary Edema<br>[ ] Pneumonia	[ ] Thoracentesis<br>[ ] Chest Tube<br>[ ] Endotracheal Intubation<br>[ ] None of the above
**c. Gastrointestinal (GI)**	[ ] Biliary<br>[ ] Peritoneal Fluid<br>[ ] FAST<br>[ ] Hernia<br>[ ] Small Bowel Obstruction	[ ] Appendicitis<br>[ ] Pneumoperitoneum<br>[ ] Paracentesis<br>[ ] Liver Biopsy<br>[ ] None of the above
**d. Urinary**	[ ] Hydronephrosis<br>[ ] Nephrolithiasis<br>[ ] Urinary Retention<br>[ ] Bladder<br>[ ] Prostate	[ ] Prostate Biopsy<br>[ ] Suprapubic Catheter<br>[ ] Nephrostomy Tube<br>[ ] None of the above
**e. Gynecological**	[ ] Intrauterine Pregnancy<br>[ ] Uterus<br>[ ] Ovaries	[ ] Intrauterine Device (IUD) Insertion<br>[ ] None of the above
**f. Vascular**	[ ] Abdominal Aortic Aneurism (AAA)<br>[ ] Deep Vein Thrombosis (DVT)<br>[ ] Arterial Flow<br>[ ] Peripheral IV Access<br>[ ] Central Line Placement	[ ] Peripherally Inserted Central Catheter (PICC) Placement<br>[ ] Arterial Line Placement<br>[ ] Intravascular Ultrasound<br>[ ] None of the above
**g. Ocular/Neurologic**	[ ] Optic Nerve Sheath Diameter<br>[ ] Eye – Posterior Chamber (i.e., retinal detachment, vitreous detachment, etc.)	[ ] Peripheral Nerve Blocks<br>[ ] Not of the above

Question 3 (continued)

**h. Musculoskeletal/Soft Tissue**	[ ] Fractures<br>[ ] Tendinopathies<br>[ ] Shoulder/Rotator Cuff<br>[ ] Cellulitis<br>[ ] Foreign Body<br>[ ] Abscess<br>[ ] Joint Effusion<br>[ ] Synovitis<br>[ ] Bursitis	[ ] Lymph Nodes [<br>] Joint Injection [<br>] Bursa Injection<br>[ ] Tendon Injection<br>[ ] Foreign Body Removal<br>[ ] Abscess Drainage<br>[ ] Arthrocentesis<br>[ ] Lymph Node Biopsy<br>[ ] None of the above
**i. Other**	[ ] Thyroid Gland<br>[ ] Parathyroid Glands<br>[ ] Neck Mass<br>[ ] Other *diagnostic training modules*, please specify: ______________	[ ] Thyroid Biopsy<br>[ ] Breast Biopsy<br>[ ] Lumbar Puncture<br>[ ] Other *procedural training modules*, please specify: ______________<br>[ ] None of the above

2. What are the barriers to use of POCUS in your facility?
   (check all that apply)
   [ ] Lack of trained providers
   [ ] Lack of ultrasound equipment
   [ ] Lack of funding for provider time for training
   [ ] Lack of funding for staff time for training
   [ ] Lack of funding for simulation space
   [ ] Lack of funding for travel
   [ ] Lack of funding for ultrasound equipment
   [ ] Lack of training opportunities
   [ ] Lack of privileging criteria
   [ ] Lack of image archiving
   [ ] Lack of standard reporting form
   [ ] No clinician champion
   [ ] Lack of facility leadership support
   [ ] Lack of service/section leadership support
   [ ] No perceived benefit to using POCUS
   [ ] Other, please specify: ____________________
   [ ] No barriers

3. Does your facility have policies regarding use of POCUS?
   ( ) Yes
   ( ) No
   If yes,
   a. Specify which policies are in place at your facility:
      (check all that apply)
      [ ] Policies regarding use by clinicians (i.e., non-radiologists, non -cardiologists)
      [ ] Policies regarding ultrasound equipment maintenance
      [ ] Policies regarding documentation
      [ ] Policies regarding image archiving
      [ ] Policies regarding privileging of clinicians
      [ ] Policies regarding supervision of trainees

Competency

4. Does your facility have a formal credentialing/privileging process to perform specific POCUS exams?
   ( ) Yes
   ( ) No

5. Does your facility require ***initial*** demonstration of individual provider competency in POCUS to grant privileges?
   ( ) Yes
   ( ) No

If yes,

a. Choose the initial competency demonstration methods:
   (check all that apply)
   [ ] Attestation/verification of basic competency obtained during graduate medical education training program (Residency/Fellowship)
   [ ] Completion of an accredited POCUS continuing medical education (CME) course with hands-on sessions
   [ ] Demonstrated/tracked at academic affiliate
   [ ] Documented minimum number of cases (e.g., 25 cases per application)
   [ ] Focused Professional Practice Evaluation (FPPE) (Prospective, Concurrent, Retrospective)
   [ ] Live proctoring
   [ ] Personal attestation of basic competency
   [ ] Simulation demonstration
   [ ] Other, please specify: ______________________________

6. Does your facility require ***ongoing*** demonstration of individual provider competency in POCUS to maintain privileges?
   ( ) Yes
   ( ) No

   If yes,

   a. Choose the demonstration methods of providers' competency:
      (check all that apply)
      [ ] Attestation/verification of competency from supervisor/service chief
      [ ] Completed/tracked at academic affiliate
      [ ] Documented minimum number of cases per reappointment cycle (e.g., 5 cases per application)
      [ ] Live proctoring
      [ ] Ongoing Professional Practice Evaluation (OPPE) (Prospective, Concurrent, Retrospective)
      [ ] Personal attestation of competency
      [ ] Simulation demonstration
      [ ] Other, please specify: __________________________

Travel/Training Support

7. Would you be in favor of physicians attending a National VA POCUS course (e.g., a 3-day National VA POCUS course in Orlando at the VA National Simulation Center or a regional VA simulation center)?
   ( ) Yes
   ( ) No

   If yes,

   a. What type of training opportunities currently exist at or nearby your facility?
      (check all that apply)
      [ ] Onsite CME course
      [ ] Offsite CME course sponsored by VA
      [ ] Non-VA sponsored (e.g., academic affiliation, self-sponsored, corporate sponsor)
      [ ] Other, please specify: ___________________________
      [ ] I don't know

Question 8 - Specific provisions would be required:

1) Educational space for lectures and hands-on practice,
2) Live models (may be volunteers or paid human ultrasound models), and
3) Ultrasound machines (either owned or loaned).

8. Would you support an onsite POCUS training course for physicians at your facility?
   (choose one)
   ( ) Yes
   ( ) No
   ( ) Maybe, please explain____

Clinical Services

9. Select all of the clinical services or units that exist in your facility.

(Person who will actually complete the follow-up Service or Section Chief survey.)

(check all that apply)	***Service or Section Chief/Designee Name***	**VA Email**	**Phone Number (including area code)**	**Extension**
[ ] MICU				
[ ] SICU				
[ ] CCU				
[ ] MICU & CCU				
[ ] MICU & SICU (Mixed)				
[ ] Specialty ICU (Includes Transplant)				
[ ] Emergency Department				
[ ] Urgent Care				
[ ] Hospital Medicine				
[ ] Acute Care Medicine				
[ ] Anesthesiology				
[ ] Dermatology				
[ ] Endocrinology				
[ ] Eye/Ophthalmology				
[ ] Gastroenterology				
[ ] Geriatrics & Extended Care				
[ ] Infectious Disease				
[ ] Nephrology				
[ ] Neurology				
[ ] Neurosurgery				
[ ] Oncology				
[ ] Orthopedics				
[ ] Pain Management – General				
[ ] Physical Medicine and Rehabilitation (PM&R)				
[ ] Pathology & Laboratory Medicine				
[ ] Podiatry				
[ ] Primary Care				
[ ] Pulmonary				
[ ] Rheumatology				
[ ] Surgery (General)				
[ ] Spinal Cord Injury				
[ ] Urology				
[ ] Vascular Surgery				
[ ] Women's Health				
[ ] None				

10. Please provide any additional comments or clarification about your survey responses here. *(optional)*

____

____

____

Thank you for your time and cooperation.

Please direct any questions to Edward O'Brien, Management Analyst, or Brandy Drum, Project Manager, HAIG, at 414-384-2000, Ext. 42354.
