# Supplementary material for: Current Use and Barriers to POCUS in Women's Health: A National Survey of Veterans Affairs Medical Centers
Source: POCUS J. 2026 Apr 22;11(1):104–9. doi: 10.24908/pocusj.v11i01.19484 (PMC13161786; doi:10.24908/pocusj.v11i01.19484)
Supplement: Supplementary file 2 [file pocusj-11-01-19484-s002.pdf]

## Supplementary File 2 - POCUS Survey for Service or Section Chief

# 2019 VHA Point-of-Care Ultrasound (POCUS) Survey – Service or Section Chief

The Office of Patient Care Services periodically surveys the programs it administers to obtain information that is, or may be of, importance to stakeholders not readily available through other means.

> ***Point-of-Care Ultrasound (POCUS) definition:*** *"Point-of-Care Ultrasound is defined as a goal-directed, bedside ultrasound examination performed by a healthcare provider to answer a specific diagnostic question or to guide performance of an invasive procedure." – Reference: Point-of-Care Ultrasound, 1st edition.*

The purpose of this survey is to assess the current state of POCUS in acute care settings in Veterans Health Administration (VHA), assess POCUS training needs across specialties, and compare data, and explore relationships that may be related to POCUS.

This survey takes approximately 15 minutes to complete online but will take additional time to gather the appropriate information using this worksheet.

***Point-of-Care Ultrasound (POCUS) definition:*** *"Point-of-Care Ultrasound is defined as a goal-directed, bedside ultrasound examination performed by a healthcare provider to answer a specific diagnostic question or to guide performance of an invasive procedure." – Reference: Point-of-Care Ultrasound, 1st edition.*

1. Is **Point-of-Care Ultrasound** used in your Service or Section?
   ( ) Yes
   ( ) No
   *If yes,*
   a. How many physicians deliver direct patient care within your Service or Section?
   ____________ (Head Count)

      a1. Of the **total** physicians that deliver direct patient care, estimate the percentage that use ANY procedural or diagnostic POCUS applications. _______ (%)

   b. Select the **diagnostic and procedural** POCUS applications routinely used by your Service or Section *and then* estimate the percentage of physicians that currently use POCUS for the diagnostic and procedural applications chosen.

( ) Few 1-25% ( ) Some 26-50% ( ) Many 51-75% ( ) Most 76-99% ( ) All 100%

| | choose all that apply | b1. % of Physicians | choose all that apply | b1. % of Physicians |
|---|---|---|---|---|
| **Cardiac** | [ ] Pericardial Effusion | _____ % | [ ] Advanced Hemodynamic Measurements (e.g., cardiac output, stroke volume) | _____ % |
| | [ ] Left Ventricular Function | _____ % | [ ] Pericardiocentesis | _____ % |
| | [ ] Venous Mapping | _____ % | | |
| | [ ] Volume Status (Inferior Vena Cava (IVC)/Internal Jugular (IJ)) | _____ % | | |
| **Pulmonary** | [ ] Pleural Effusion | _____ % | [ ] Thoracentesis | _____ % |
| | [ ] Pneumothorax | _____ % | [ ] Chest Tube | _____ % |
| | [ ] Pulmonary Edema | _____ % | [ ] Endotracheal Intubation | _____ % |
| | [ ] Pneumonia | _____ % | | |
| **Gastrointestinal (GI)** | [ ] Biliary | _____ % | [ ] Appendicitis | _____ % |
| | [ ] Peritoneal Fluid | _____ % | [ ] Pneumoperitoneum | _____ % |
| | [ ] Focused Assessment with Sonography for Trauma (FAST) | _____ % | [ ] Paracentesis | _____ % |
| | [ ] Hernia | _____ % | [ ] Liver Biopsy | _____ % |
| | [ ] Small Bowel Obstruction | _____ % | | |
| **Urinary** | [ ] Hydronephrosis | _____ % | [ ] Prostate | _____ % |
| | [ ] Nephrolithiasis | _____ % | [ ] Prostate Biopsy | _____ % |
| | [ ] Urinary Retention | _____ % | [ ] Suprapubic Catheter | _____ % |
| | [ ] Bladder | _____ % | [ ] Nephrostomy Tube | _____ % |
| **Gynecological** | [ ] Intrauterine Pregnancy | _____ % | [ ] Ovaries | _____ % |
| | [ ] Uterus | _____ % | [ ] Intrauterine Device (IUD) Insertion | _____ % |
| **Vascular** | [ ] Abdominal Aortic Aneurism (AAA) | _____ % | [ ] Central Line Placement | _____ % |
| | [ ] Deep Vein Thrombosis (DVT) | _____ % | [ ] Peripherally Inserted Central Catheter (PICC) Placement | _____ % |
| | [ ] Arterial Flow | _____ % | [ ] Arterial Line Placement | _____ % |
| | [ ] Peripheral IV Access | _____ % | [ ] Intravascular Ultrasound | _____ % |

Question 1b. (continued)

( ) Few 1-25% ( ) Some 26-50% ( ) Many 51-75% ( ) Most 76-99% ( ) All 100%

| | choose all that apply | b1. % of Physicians | choose all that apply | b1. % of Physicians |
|---|---|---|---|---|
| **Ocular/ Neurologic** | [ ] Optic Nerve Sheath Diameter<br>[ ] Eye – Posterior Chamber (i.e., retinal detachment, vitreous detachment, etc.) | _____ %<br>_____ % | [ ] Peripheral Nerve Blocks | _____ % |
| **Musculoskeletal/ Soft Tissue** | [ ] Fractures<br>[ ] Tendinopathies<br>[ ] Shoulder/Rotator Cuff<br>[ ] Cellulitis<br>[ ] Foreign Body<br>[ ] Abscess<br>[ ] Joint Effusion<br>[ ] Synovitis<br>[ ] Bursitis | _____ %<br>_____ %<br>_____ %<br>_____ %<br>_____ %<br>_____ %<br>_____ %<br>_____ %<br>_____ % | [ ] Lymph Nodes<br>[ ] Joint Injection<br>[ ] Bursa Injection<br>[ ] Tendon Injection<br>[ ] Foreign Body Removal<br>[ ] Abscess Drainage<br>[ ] Arthrocentesis<br>[ ] Lymph Node Biopsy | _____ %<br>_____ %<br>_____ %<br>_____ %<br>_____ %<br>_____ %<br>_____ %<br>_____ % |
| **Other** | [ ] Thyroid Gland<br>[ ] Parathyroid Glands<br>[ ] Neck Mass<br>[ ] Other ***diagnostic*** *applications used*, please specify: ______________ | _____ %<br>_____ %<br>_____ %<br>_____ % | [ ] Thyroid Biopsy<br>[ ] Breast Biopsy<br>[ ] Lumbar Puncture<br>[ ] Other ***procedural*** *applications used*, please specify: _____________ | _____ %<br>_____ %<br>_____ %<br>_____ % |

**POCUS Barriers**

2. What are the barriers to the use of POCUS in your Service or Section?
(check all that apply)
[ ] Lack of trained Physicians
[ ] Lack of ultrasound equipment
[ ] Lack of funding for physician time for training
[ ] Lack of funding for support staff time for training
[ ] Lack of funding for simulation space
[ ] Lack of funding for travel
[ ] Lack of funding for ultrasound equipment
[ ] Lack of training opportunities
[ ] Lack of privileging criteria
[ ] Lack of image archiving
[ ] Lack of standard reporting form
[ ] No clinician champion
[ ] Lack of facility leadership support
[ ] Lack of service/section leadership support
[ ] No perceived benefit to using POCUS
[ ] Other, please specify: _______________
[ ] No barriers

**Diagnostic and Procedural Training Modules**

3. Is POCUS training desired by physicians in your Service or Section? *(If 'No' to Q1 answer Q3 and skip to Q5)*
( ) Yes
( ) No

*If yes,*

a. For each body system below, select the diagnostic and procedural POCUS training modules your staff could benefit from: (check all that apply for each body system)

| Body System | | |
|---|---|---|
| **Cardiac** | [ ] Pericardial Effusion<br>[ ] Left Ventricular Function<br>[ ] Venous Mapping<br>[ ] Volume Status (Inferior Vena Cava (IVC)/Internal Jugular (IJ)) | [ ] Advanced Hemodynamic Measurements (e.g., cardiac output, stroke volume)<br>[ ] Pericardiocentesis |
| **Pulmonary** | [ ] Pleural Effusion<br>[ ] Pneumothorax<br>[ ] Pulmonary Edema<br>[ ] Pneumonia | [ ] Thoracentesis<br>[ ] Chest Tube<br>[ ] Endotracheal Intubation |
| **Gastrointestinal (GI)** | [ ] Biliary<br>[ ] Peritoneal Fluid<br>[ ] FAST<br>[ ] Hernia<br>[ ] Small Bowel Obstruction | [ ] Appendicitis<br>[ ] Pneumoperitoneum<br>[ ] Paracentesis<br>[ ] Liver Biopsy |
| **Urinary** | [ ] Hydronephrosis<br>[ ] Nephrolithiasis<br>[ ] Urinary Retention<br>[ ] Bladder | [ ] Prostate<br>[ ] Prostate Biopsy<br>[ ] Suprapubic Catheter<br>[ ] Nephrostomy Tube |
| **GYN** | [ ] Intrauterine Pregnancy<br>[ ] Uterus<br>[ ] Ovaries | [ ] Intrauterine Device (IUD) Insertion |
| **Vascular** | [ ] Abdominal Aortic Aneurism (AAA)<br>[ ] Deep Vein Thrombosis (DVT)<br>[ ] Arterial Flow<br>[ ] Peripheral IV Access | [ ] Central Line Placement<br>[ ] Peripherally Inserted Central Catheter (PICC) Placement<br>[ ] Arterial Line Placement<br>[ ] Intravascular Ultrasound |
| **Ocular/Neurologic** | [ ] Optic Nerve Sheath Diameter<br>[ ] Eye – Posterior Chamber (i.e., retinal detachment, vitreous detachment, etc.) | [ ] Peripheral Nerve Blocks |
| **Musculoskeletal/Soft Tissue** | [ ] Fractures<br>[ ] Tendinopathies<br>[ ] Shoulder/Rotator Cuff<br>[ ] Cellulitis<br>[ ] Foreign Body<br>[ ] Abscess<br>[ ] Joint Effusion<br>[ ] Synovitis<br>[ ] Bursitis | [ ] Lymph Nodes [<br>] Joint Injection [<br>] Bursa Injection<br>[ ] Tendon Injection<br>[ ] Foreign Body Removal<br>[ ] Abscess Drainage<br>[ ] Arthrocentesis<br>[ ] Lymph Node Biopsy |
| **Other** | [ ] Thyroid Gland<br>[ ] Parathyroid Glands<br>[ ] Neck Mass<br>[ ] Other *diagnostic training modules*, please specify: | [ ] Thyroid Biopsy<br>[ ] Breast Biopsy<br>[ ] Lumbar Puncture<br>[ ] Other *procedural training modules*, please specify: |

**POCUS Brands used**

4. What brand of ultrasound machine(s) is/are being used for POCUS within your Service or Section?

| (check all that apply) | a. Indicate the type of device: (choose all that apply for selected units)<br>[ ] **H**andheld (i.e., pocket device)<br>[ ] **S**mall Cart based (i.e., laptop style)<br>[ ] **L**arge Cart based (i.e., radiology style) | | |
|---|---|---|---|
| [ ] Aloka | H | S | L |
| [ ] Bard | H | S | L |
| [ ] BK Medical | H | S | L |
| [ ] Butterfly IQ | H | S | L |
| [ ] Clarius | H | S | L |
| [ ] Ellex | H | S | L |
| [ ] Esaote | H | S | L |
| [ ] GE | H | S | L |
| [ ] Hitachi | H | S | L |
| [ ] Mindray | H | S | L |
| [ ] Olympus | H | S | L |
| [ ] Philips | H | S | L |
| [ ] Samsung | H | S | L |
| [ ] Siemens | H | S | L |
| [ ] SiteRite | H | S | L |
| [ ] Sonomet | H | S | L |
| [ ] Sonosite/Fujifilm | H | S | L |
| [ ] Terason | H | S | L |
| [ ] Toshiba | H | S | L |
| [ ] Verathon | H | S | L |
| [ ] Zonare | H | S | L |
| [ ] Brand Unknown | H | S | L |
| [ ] None *(continue to Q5)* | | | |

**Device types by POCUS brand and number available**

*For each Brand and Type of Device chosen,*

4a1. Indicate the number of ultrasound machines that are dedicated/shared/provided by physician or facility:

| *(Do not count any unit more than once.)* Complete for each Device Type Chosen (check all that apply) | | (Number of Units – If none, enter '0')<br>Dedicated to this Service or Section | Shared with other departments | Provided by physician | Provided by facility |
|---|---|---|---|---|---|
| Aloka | Handheld (i.e., pocket device) | | | | |
| | Small Cart based (i.e., laptop style) | | | | |
| | Large Cart based (i.e., radiology style) | | | | |
| Bard | Handheld (i.e., pocket device) | | | | |
| | Small Cart based (i.e., laptop style) | | | | |
| | Large Cart based (i.e., radiology style) | | | | |
| BK Medical | Handheld (i.e., pocket device) | | | | |
| | Small Cart based (i.e., laptop style) | | | | |
| | Large Cart based (i.e., radiology style) | | | | |

Question 4a1. (continued)

| Complete for each Device Type Chosen (check all that apply) | | (Number of Units – If none, enter '0') (Do not count any unit more than once.) Dedicated to this Service or Section | Shared with other departments | Provided by practitioner | Provided by facility |
|---|---|---|---|---|---|
| Butterfly IQ | Handheld (i.e., pocket device) | | | | |
| | Small Cart based (i.e., laptop style) | | | | |
| | Large Cart based (i.e., radiology style) | | | | |
| Clarius | Handheld (i.e., pocket device) | | | | |
| | Small Cart based (i.e., laptop style) | | | | |
| | Large Cart based (i.e., radiology style) | | | | |
| Ellex | Handheld (i.e., pocket device) | | | | |
| | Small Cart based (i.e., laptop style) | | | | |
| | Large Cart based (i.e., radiology style) | | | | |
| Esaote | Handheld (i.e., pocket device) | | | | |
| | Small Cart based (i.e., laptop style) | | | | |
| | Large Cart based (i.e., radiology style) | | | | |
| GE | Handheld (i.e., pocket device) | | | | |
| | Small Cart based (i.e., laptop style) | | | | |
| | Large Cart based (i.e., radiology style) | | | | |
| Hitachi | Handheld (i.e., pocket device) | | | | |
| | Small Cart based (i.e., laptop style) | | | | |
| | Large Cart based (i.e., radiology style) | | | | |
| Mindray | Handheld (i.e., pocket device) | | | | |
| | Small Cart based (i.e., laptop style) | | | | |
| | Large Cart based (i.e., radiology style) | | | | |
| Olympus | Handheld (i.e., pocket device) | | | | |
| | Small Cart based (i.e., laptop style) | | | | |
| | Large Cart based (i.e., radiology style) | | | | |
| Philips | Handheld (i.e., pocket device) | | | | |
| | Small Cart based (i.e., laptop style) | | | | |
| | Large Cart based (i.e., radiology style) | | | | |
| Samsung | Handheld (i.e., pocket device) | | | | |
| | Small Cart based (i.e., laptop style) | | | | |
| | Large Cart based (i.e., radiology style) | | | | |
| Siemens | Handheld (i.e., pocket device) | | | | |
| | Small Cart based (i.e., laptop style) | | | | |
| | Large Cart based (i.e., radiology style) | | | | |
| SiteRite | Handheld (i.e., pocket device) | | | | |
| | Small Cart based (i.e., laptop style) | | | | |
| | Large Cart based (i.e., radiology style) | | | | |
| Sonomet | Handheld (i.e., pocket device) | | | | |
| | Small Cart based (i.e., laptop style) | | | | |
| | Large Cart based (i.e., radiology style) | | | | |

Question 4a1. (continued)

(Number of Units – If none, enter '0')
(Do not count any unit more than once.)

| Complete for each Device Type Chosen (check all that apply) | | Dedicated to this Service or Section | Shared with other departments | Provided by practitioner | Provided by facility |
|---|---|---|---|---|---|
| Sonosite/ Fujufilm | Handheld (i.e., pocket device) | | | | |
| | Small Cart based (i.e., laptop style) | | | | |
| | Large Cart based (i.e., radiology style) | | | | |
| Terason | Handheld (i.e., pocket device) | | | | |
| | [Small Cart based (i.e., laptop style) | | | | |
| | Large Cart based (i.e., radiology style) | | | | |
| Toshiba | Handheld (i.e., pocket device) | | | | |
| | Small Cart based (i.e., laptop style) | | | | |
| | Large Cart based (i.e., radiology style) | | | | |
| Verathon | Handheld (i.e., pocket device) | | | | |
| | Small Cart based (i.e., laptop style) | | | | |
| | Large Cart based (i.e., radiology style) | | | | |
| Zonare | Handheld (i.e., pocket device) | | | | |
| | Small Cart based (i.e., laptop style) | | | | |
| | Large Cart based (i.e., radiology style) | | | | |
| Brand Unknown | Handheld (i.e., pocket device) | | | | |
| | Small Cart based (i.e., laptop style) | | | | |
| | Large Cart based (i.e., radiology style) | | | | |

**POCUS Policies**

5. Are there **facility-wide** policies regarding use of POCUS? *(If 'No' to Q1 answer Q5 then skip to Q14)*
   ( ) Yes
   ( ) No

   *If yes,*
   a. Specify which policies are in place at your facility.
      (check all that apply)
      [ ] Policies regarding use by clinicians
      [ ] Policies regarding ultrasound equipment maintenance
      [ ] Policies regarding documentation
      [ ] Policies regarding image archiving
      [ ] Policies regarding privileging of clinicians
      [ ] I'm not sure

6. Does your Service or Section have its own specific policies regarding use of POCUS?
   ( ) Yes
   ( ) No

   *If yes,*
   a. Specify which policies are in place for your Service or Section.
      (check all that apply)
      [ ] Policies regarding use by clinicians
      [ ] Policies regarding ultrasound equipment maintenance
      [ ] Policies regarding documentation
      [ ] Policies regarding image archiving
      [ ] Policies regarding privileging of clinicians
      [ ] Other, please specify: ______________________________
      [ ] I'm not sure

**Quality Assurance, Trainees**

7. Do trainees rotate with Physicians in your Service or Section?
( ) Yes
( ) No

*If yes,*

a. Specify type(s) of trainees.
(check all that apply)
[ ] Medical students
[ ] Residents
[ ] Fellows
[ ] Nursing students
[ ] Physician Assistant (PA) students
[ ] Advance Practice Registered Nurse (APRN) Students
[ ] Other

b. For which of these trainee types does your Service or Section provide POCUS training?
(check all that apply)
[ ] Medical students
[ ] Residents
[ ] Fellows
[ ] Nursing students
[ ] PA students
[ ] APRN students
[ ] Other
[ ] None of the above

**POCUS Documentation/Workload**

8. Are POCUS images recorded and saved within your Service or Section?
(choose one)
( ) Yes
( ) No
( ) I don't know

*If yes,*

a. Where are the POCUS images saved?
(check all that apply)
[ ] On the machine
[ ] Electronic medical record
[ ] VHA server
[ ] Non-VHA server
[ ] Other, please specify: ______________________
[ ] I don't know

b. Are these recordings reviewed by a second level, (i.e., POCUS designee/expert)?
(choose one)
( ) Yes
( ) No
( ) I don't know

c. Is there a protocol or agreement for preliminary vs. official interpretation?
(choose one)
( ) Yes
( ) No
( ) I don't know

9. Does your Service or Section perform periodic quality assurance (QA) reviews of POCUS cases?
   (choose one)
   ( ) Yes
   ( ) No
   ( ) I don't know
   *If yes,*
   a. Choose the review methods:
      (check all that apply)
      [ ] Retrospective review of cases
      [ ] Demonstration of skills using live models
      [ ] Demonstration of skills using simulation
      [ ] Other, please specify: ____________________
   *If no or I don't know,*
   b. Would you be interested in establishing a QA program in your Service or Section?
      (choose one)
      ( ) Yes
      ( ) No
      ( ) I'm not sure at this time

10. How is workload captured for POCUS in your Service or Section?
    (check all that apply)
    [ ] Encounters are generated and Physicians select CPT codes
    [ ] Coders review documentation and select appropriate codes for encounters
    [ ] Workload is not captured for POCUS
    [ ] Other, please specify: ______________________
    [ ] I don't know

**Training**

11. What percentage of physicians in your Service or Section have had formal training through a continuing medical education (CME) workshop in POCUS?
    (choose one)
    ( ) None (0%)
    ( ) Few (1-25%)
    ( ) Some (26-50%)
    ( ) Many (51-75%)
    ( ) Most (76-99%)
    ( ) All (100%)
    ( ) I don't know

12. What percentage of physicians in your Service or Section received POCUS training as part of their residency or fellowship training?
    (choose one)
    ( ) None (0%)
    ( ) Few (1-25%)
    ( ) Some (26-50%)
    ( ) Many (51-75%)
    ( ) Most (76-99%)
    ( ) All (100%)
    ( ) I don't know

13. What percentage of physicians in your Service or Section have completed a separate ultrasound or POCUS fellowship?
(choose one)
( ) None (0%)
( ) Few (1-25%)
( ) Some (26-50%)
( ) Many (51-75%)
( ) Most (76-99%)
( ) All (100%)
( ) I don't know

**POCUS Process for Training**

14. Does your facility have a process to obtain or provide POCUS training for the physicians that desire training in your Service or Section?
( ) Yes
( ) No
*If yes,*
a. What type of training opportunities currently exist at or nearby your facility?
(check all that apply)
[ ] Onsite CME course
[ ] Offsite CME course sponsored by VHA
[ ] Non-VHA sponsored (e.g., academic affiliation, self-sponsored, corporate sponsor)
[ ] Other, please specify: ____________________________
[ ] I don't know

**Travel Support and Comments**

15. Is your VHA facility's Simulation Center/Program currently offering POCUS training?
(choose one)
( ) Yes
( ) No
( ) No center/program exists at our facility
( ) I don't know

16. Would you be in favor of physicians in your Service or Section attending a National VHA POCUS course (e.g., a 3-day National VA POCUS course in Orlando at the VA National Simulation Center or a regional VA simulation center)?
(choose one)
( ) Yes
( ) No
*If yes,*
a. For physicians in your Service or Section that want to attend a National VHA POCUS course, which of the following would you support?
(choose all that apply)
[ ] Cost of travel
[ ] Advocate for facility to fund travel
[ ] Release from clinical duties
[ ] Other, please specify: ___________________________

When answering question 17 please take into consideration these specific provisions that would be required for onsite POCUS training.
1) Educational space for lectures and hands-on practice,
2) Live models (may be volunteers or paid human ultrasound models), and
3) Ultrasound machines (either owned or loaned).

17. Would you support an onsite POCUS training course for physicians at your facility? (choose one)
( ) Yes
( ) No
( ) Maybe, please explain: _______________

18. Please provide any additional comments or clarification about your survey responses here. (optional)
________________________________________________________________________________

***Thank you for your time and cooperation.***

**Please direct any questions to Edward O'Brien, Program Analyst, or Brandy Drum, Project Manager, HAIG, at 414-384-2000, Ext. 42354.**
